# Zigzag Turning Preference of Freely Crawling Cells

**DOI:** 10.1371/journal.pone.0020255

**Published:** 2011-06-07

**Authors:** Taeseok Daniel Yang, Jin-Sung Park, Youngwoon Choi, Wonshik Choi, Tae-Wook Ko, Kyoung J. Lee

**Affiliations:** 1 Department of Physics, Korea University, Seoul, Korea; 2 Center for Cell Dynamics, Korea University, Seoul, Korea; 3 National Institute for Mathematical Sciences, Daejeon, Korea; University of Manchester, United Kingdom

## Abstract

The coordinated motion of a cell is fundamental to many important biological processes such as development, wound healing, and phagocytosis. For eukaryotic cells, such as amoebae or animal cells, the cell motility is based on crawling and involves a complex set of internal biochemical events. A recent study reported very interesting crawling behavior of single cell amoeba: in the absence of an external cue, free amoebae move randomly with a noisy, yet, discernible sequence of ‘run-and-turns’ analogous to the ‘run-and-tumbles’ of swimming bacteria. Interestingly, amoeboid trajectories favor zigzag turns. In other words, the cells bias their crawling by making a turn in the opposite direction to a previous turn. This property enhances the long range directional persistence of the moving trajectories. This study proposes that such a zigzag crawling behavior can be a general property of any crawling cells by demonstrating that 1) microglia, which are the immune cells of the brain, and 2) a simple rule-based model cell, which incorporates the actual biochemistry and mechanics behind cell crawling, both exhibit similar type of crawling behavior. Almost all legged animals walk by alternating their feet. Similarly, all crawling cells appear to move forward by alternating the direction of their movement, even though the regularity and degree of zigzag preference vary from one type to the other.

## Introduction

The crawling of cells plays a key role in biological development, wound healing, metastasis of cancer cells, and many other physiological and pathological processes. The process involves the complex coordination of a range of molecular events, including directed assembly of actin monomers, gelation process of actin filaments, formation of focal adhesion sites, disassembly of crosslinked network of actin filaments, and recycling monomeric actins [5], [21], [26]. The nexus of these molecular actions is also coupled to the cell's sensory systems, which recognize and interpret the various external cues from the environment. Over many years, significant advances have been made in identifying the biochemical components responsible for the individual molecular events, but how they are coordinated and translated into the behavior of cell migration is not completely understood [12], [14], [18], [24], [28].

In many cases cell motility is driven by external cues, such as spatial or temporal modulations of attractants (or repellents) [9], [11]. However, cells even crawl in ‘darkness’ (i.e, in the absence of external gradients), e.g. to detect harmful invaders or search for food. In a natural situation, this cell-intrinsic motility might simultaneously coexist with the directed motions driven by extrinsic factors. Of the two different origins, one may dominate over the other or both may play a significant role.

Over many years of biological evolution, cells presumably have developed some special crawling strategies. The existence of optimal searching strategies in animal populations has been tested and modeled in a number of different circumstances [2], [7], [10], [23], but there are few reports on crawling single cells [8], [20]. Only a few years ago, Li *et al.* [15] reported their first experimental observations of the crawling behavior of isolated Dictyostelium discodium amoebae. They found that free amoebae in the absence of external cues crawl randomly but with a long range directional persistence. Interestingly, this long-range persistence originates in part from the existence of many small zigzag turns. The cell trajectories can be viewed as a sequence of small ‘runs’ (more or less, straight movements) and ‘turns,’ similar to the ‘run-and-tumble’ motion of petritrichously flagellated E. Coli bacteria [3] or that of biflagellated alga Chlamydomonas [19]. However, there is a major difference between dicty cells and bacteria or Chlamydomonas. While the turning events of bacteria and Chlamydomonas are purely stochastic, those of amoebae exhibit short-term memory. Amoebae have a strong tendency to turn away from a previous turn. This interesting finding was also confirmed by Bosgraaf and Haastert, who quantified the ordered extension of pseudopodia of amoeboid cells [4].

This study examined whether the observed zigzag crawling behavior and the long-range directional persistence of Dictyostelium amoebae can be a general property of any crawling cells. First of all, microglia, which are the immune cells of the brain, were investigated [13], [17] as another example. The free microglia in a cell culture also exhibited similar type of zigzag crawling motion. Second, a simple activator-inhibitor kinetic model [24], which incorporates some of the essential biochemical reactions of actin polymerization (and depolymerization) and cell mechanics, was used to show that freely crawling cells intrinsically support zigzag turns and that the degree of the zigzag turning preference can be tuned by changing some key parameters associated with the actin polymerization process.

## Results

### Directional persistence in crawling microglia trajectories

**Figure 1:**
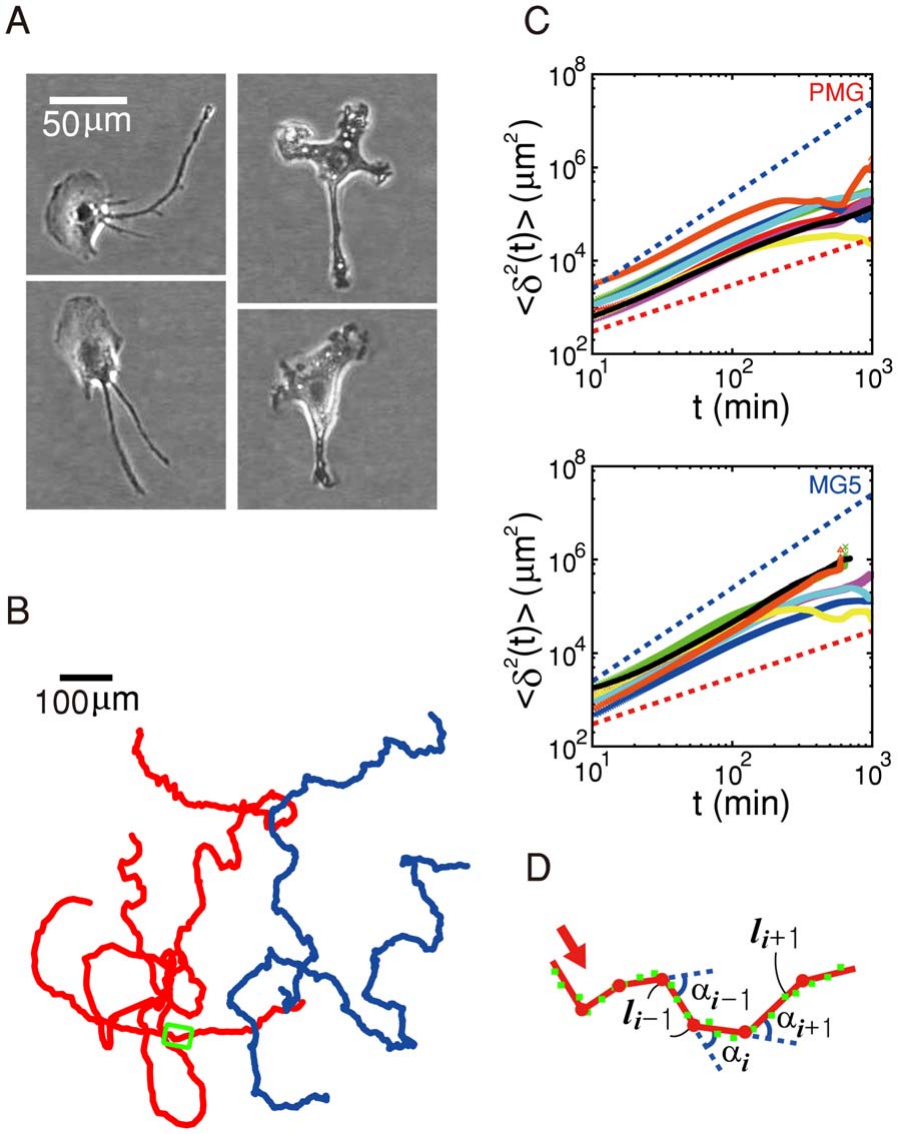
Crawling trajectories of PMG and MG5 cells. A) Snapshot images showing the typical shapes of crawling PMG cells (left) and MG5 cell line (right), B) four crawling trajectories (red, PMG, total duration 56 and 25 hrs; blue, MG5, total duration 12 and 18 hrs), C) Log-log plot of mean-squared displacements vs. time interval 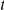 (n = 8 for each plot), D) Blown up image showing a sequence of small zigzag turns [boxed area in (B)]. The green dots are the centroid positions and the red dots mark a turning event. In (C), the slopes of the red and blue dotted lines are 1 and 2, respectively.

Figure 1 shows an image of microglia cells dissociated from rat brains (PMG: left) and mouse-derived microglia cell lines (MG5: right), which are approximately 2 days old in a culture dish. They are motile and have a fan-shaped cell body with a broad ruffling fronts and tail-like long structures in the rear end [see Movie S1]. The trajectories of many freely moving PMG and MG5 cells were monitored continuously for more than twenty hours and two representative cases are shown for each in Fig. 1B (red: PMG, blue: MG5). The individual trajectories are intrinsic to each cell because there is no externally imposed gradient guiding the cells in the very low density preparation. They move quite slowly with an average velocity 1
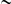
4 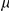
m/min.

The non-interacting PMG and MG5 cell trajectories exhibit a strong directional persistence as shown in Fig. 1C which plots the mean-square displacements 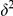 vs. time 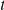 on a log-log scale for eight different cells in each case. For approximately 20
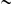
200 minutes, the graphs are almost straight with a slope in between 1 and 2 (PMG: mean 1.42, s.d. 0.08; MG5: mean 1.57, s.d. 0.11). Both PMG and MG5 cells are neither purely diffusive nor ballistic objects but crawl with a long-range directional persistence. Moreover, their mean velocity distributions over 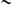
10 minutes show a non-Gaussian ‘hollow-shaped’ distribution in 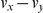 space (see Fig. S1), which is an important characteristics that excludes two well-known models of random motion for the observed PMG cell trajectories, the worm-like chain model [22] and Ornstein-Uhlenbeck (OU) model [27]. This is similar to the case of crawling Dictyostelium amoebae [15]. Qualitatively, similar characteristics were also observed with mouse-derived MG5 microglia cell lines [see Fig. 1B (blue trajectories) and Fig. 1C (bottom)]. Both PMG or MG5 cells typically have tail-like projections in their rear ends, but their shapes do not show any noticeable correlation with the moving speed or direction of the cells.

### Preference for zigzag turns

As in the case of freely moving Dictyostelium amoebae, the long-range directional persistence of crawling microglia appears to be closely related to their preference of making small zigzag turns. Under a close-up view, the trajectories followed by moving microglia cell can be viewed as a chain of small line segments 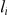 (see Fig. 1D and Movie S1). Along each segment, the cell moves more or less straight until the end, where a turning event with an angle 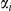 occurs (see Materials and Methods for the details on data processing).

The probability density function of the inter-turn time intervals 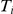 is well fitted by an exponential function except for the small T (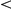
1 min) regime (see Fig. S2). Thus, the turning events may be viewed as a Poisson process. The probability density functions of the turning angles 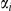, inter-turn distances 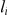, and inter-turn average velocities 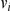 are also given in Fig. S2. The distribution of 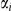 may also be viewed as two exponential functions sitting back-to-back, but for the large 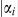 regime the fitting becomes poor. This is mainly due to the frequent ‘front splitting events’ resulting in very sharp turns near 90 [see Fig. S3(A)] and 180 turns, in which cells return to where they originated from. Table 1 lists the mean values of the quantities characterizing the cell trajectories.

**Table 1:** Summary of the characteristic values of zigzag turns.

cell type	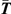 (min)	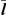 ( 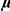 m)	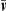 ( 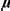 m/min)	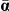 (degree)	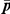	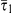 (min)	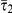 (min)
PMG (n = 8)	1.9 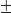 0.2	5.1 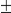 1.5	2.7 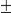 1.2	48.3 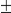 7.3	1.9 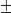 0.1	0.6 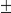 0.1	33.7 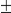 3.6
MG5 (n = 8)	2.0 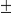 0.1	5.3 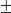 0.9	2.7 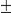 0.6	52.0 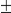 5.5	1.6 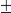 0.1	0.9 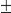 0.1	41.9 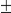 5.2
The model cell 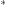	1.38 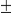 0.02	9.1 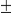 0.2	6.3 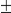 1.4	1.9 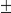 0.1	1.9 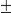 0.1	5.0 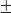 1.4	51.5 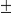 4.7
Dicty (n = 12) 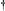	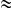 0.67	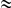 5	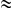 7	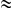 38.4	2.1 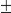 0.1	–	–

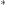
 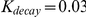, n = 10.

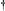
Li et al. [17].

**Figure 2:**
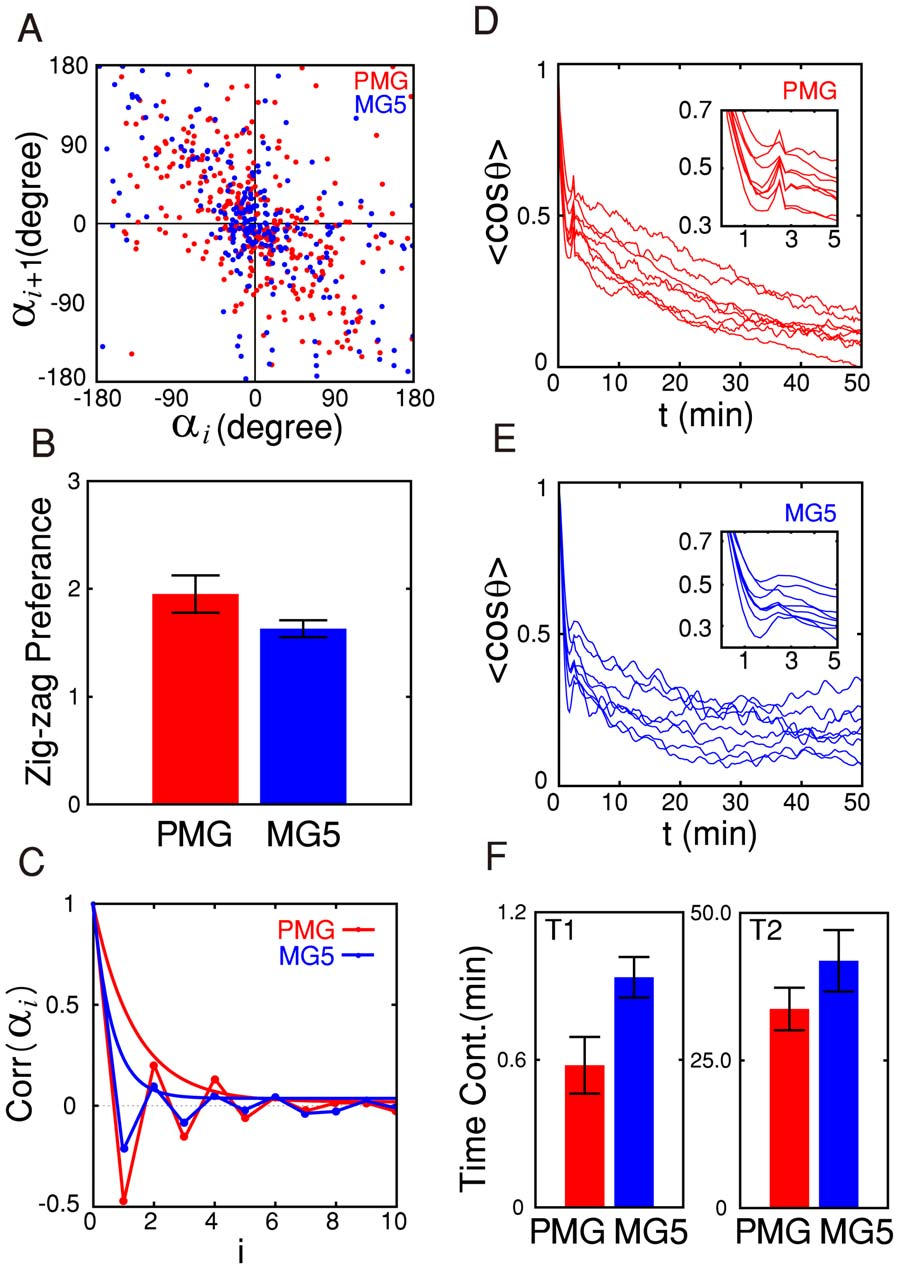
Zigzag preference of crawling PMG and MG5 cells. A) Typical return maps of turning angles 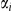 (red, PMG; blue, MG5), B) Histograms of the zigzag preference 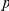 (n = 8 for each case), C) Auto-correlation functions of 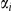 and their corresponding fits to an exponential function, D) and E) Auto-correlation functions of the instantaneous direction of movement for PMG and MG5 cells, respectively (n = 8 for each case). F) Histograms of the two time constants obtained by fitting the curves in (D) and (E) to 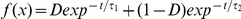. 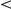
cos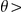 in (D) and (E) are the ensemble time average over the entire observation duration of the inner-product between the two directional unit vectors separated by 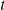.

The preference for zigzag turns is well depicted in the return map of 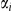, as shown in Fig. 2A, which plots the relationship of 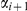 to 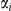. For a given example, the total number of points (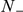) in the upper-left and lower-right quadrants combined for PMG and MG5 was 255 and 168, respectively, while the total number of points (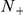) in the upper-right and lower-left quadrants combined for PMG and MG5 was 101 and 78, respectively. In other words, crawling microglia tend to make turns in the opposite direction of the previous turn with a zigzag preference index 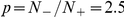 and 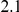 for PMG and MG5, respectively. The 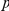 value varies from one cell to another, and MG5 cell lines show somewhat smaller values (1.6, s.d. 0.1, n = 8) than PMG cells (1.9, s.d. 0.2, n = 8) (see Fig. 2B). The dependence of 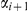 on 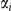 suggests the existence of a determinism or memory in the selection process of the turning angle, but the memory is noisy in that the zigzag turn is not guaranteed for every turn. Moreover, the memory is short-term, not lasting long beyond one step forward, as confirmed by the autocorrelation function of 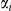 in Fig. 2C. Figures 2D (PMG) and 2E (MG5) show mean value of the dot product of two tangent vectors separated by time 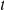, as a measure of the directional persistence. They fall quickly during the first minute or so and decay slowly beyond that point. Indeed, they can be well fitted to a sum of two exponentially decaying functions 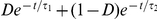 (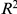 values, PMG 0.91
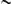
0.98, MG5 0.81
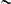
0.95), showing that the free microglia cell motility involves both a strong short-range directional correlation due to the existence of small ‘runs’ (with the time constant 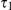) and a long-range directional persistence mediated by the zigzag turning preference (with the time constant 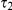).

### Zigzag turns in a mathematical model cell

Few mathematical models have discussed the mechano-chemical aspects of cell crawling, particularly for the long-term behavioral pattern of free cells. Regarding the experimental observations on the crawling pattern of amoebae, Li *et al.* proposed a simple mathematical model to describe the dynamics of the instantaneous direction of motion 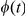 [15]. The model was basically a noise-driven damped linear oscillator that was facilitated by low frequency white noise. Although the proposed model could produce some of the essential features, such as the power spectral density function of 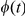 and the autocorrelation function of 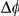 from their experimental data, it was a simple model lacking a detailed connection to the biochemical reactions governing the cell shape and crawling behavior. Recently, Nishimura *et al.* [18] proposed a more realistic model for cell locomotion and cytofission, and discussed the important role of the actin polymerization-suppression factor, known as the “cortical factor,” for determining the directional persistence of cell migration. They found that the persistence could be changed significantly by two parameters, the threshold value of actin polymerization and the rate of transferring the cortical factor from the cytosol to the cortical layer.

Another realistic mathematical model for crawling cell was developed recently by Satulovsky *et al.* [24]. It is a rule-based top-down model that incorporates some of the essential chemical and mechanical components of cell crawling. This general model was developed to understand how the signaling events controlling cell protrusion and retraction are coordinated to generate the shapes and migration patterns of different cell types. A range of migrating cells could be produced depending on the values of some key parameters, including Dictyostelium amoebae, fibroblasts, keratocytes, and neurons. In this study, the zigzag motility of this model cell was examined.

On a two dimensional surface, a simply closed loop, whose boundary can protrude by a local activation signal or retreat in response to a global inhibition signal, was modeled as a cell. The local activator 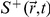 induces the polymerization of actins, which moves the cell boundary in the forward direction of movement. On the other hand, the global inhibitor, 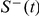, dissociates the actin network and retreats the cell boundary toward the center of the cell if 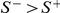. Therefore, the moving front is activator rich, whereas the tail part is inhibitor rich. The growth rate of 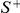 is a nonlinear function of 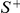 and 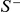, and the concentration of the inhibitor 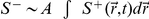, where 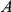 is the area enclosed by the model cell boundary. The model also includes a stochastic process for the formation (and dissociation) of focal adhesion sites: For every iterative time step, each point along the cell boundary has some likelihood of adhesion to and detachment from the substrate with a probability 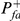 and 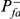, respectively.

**Figure 3:**
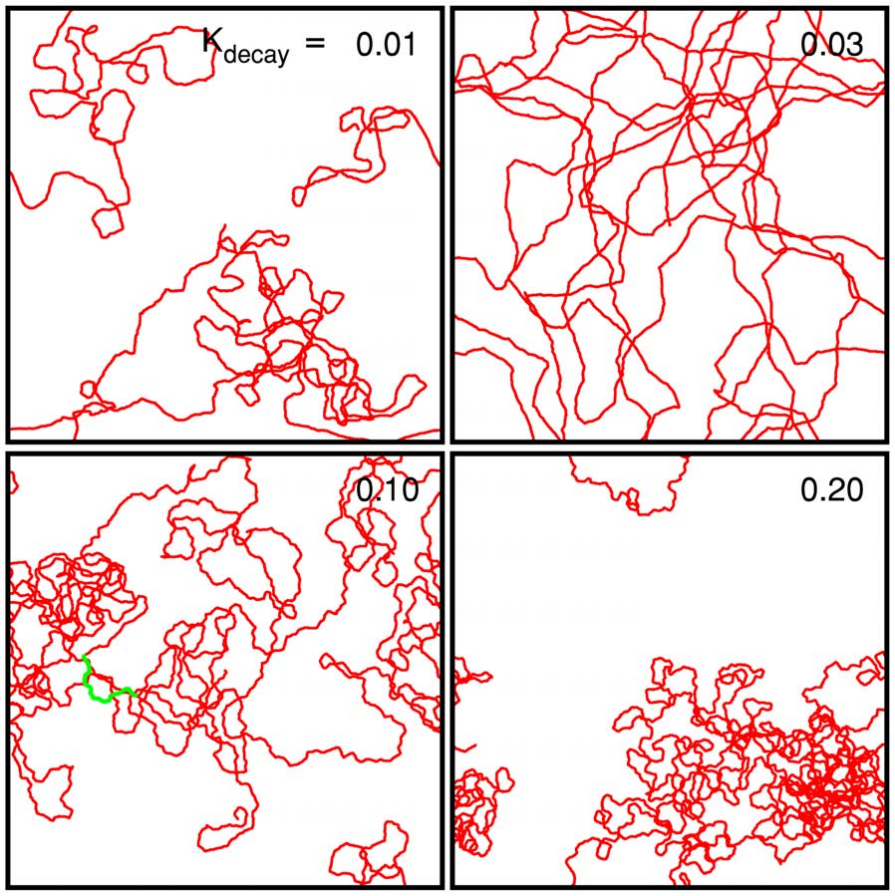
Centroid trajectories of the crawling model cells for different parameter values of 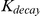. The other parameter values were fixed as follows: 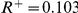 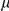
m/s, 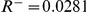 1/s, 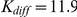 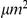/s, 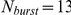, 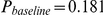 1/(s 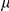
m), 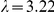 1/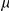
m, 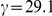 1/s, 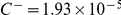 1/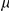
m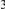, 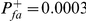, 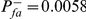, 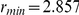 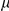
m. Each frame is 1000
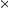
1000 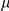
m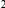 and includes 200000 iteration steps.

**Figure 4:**
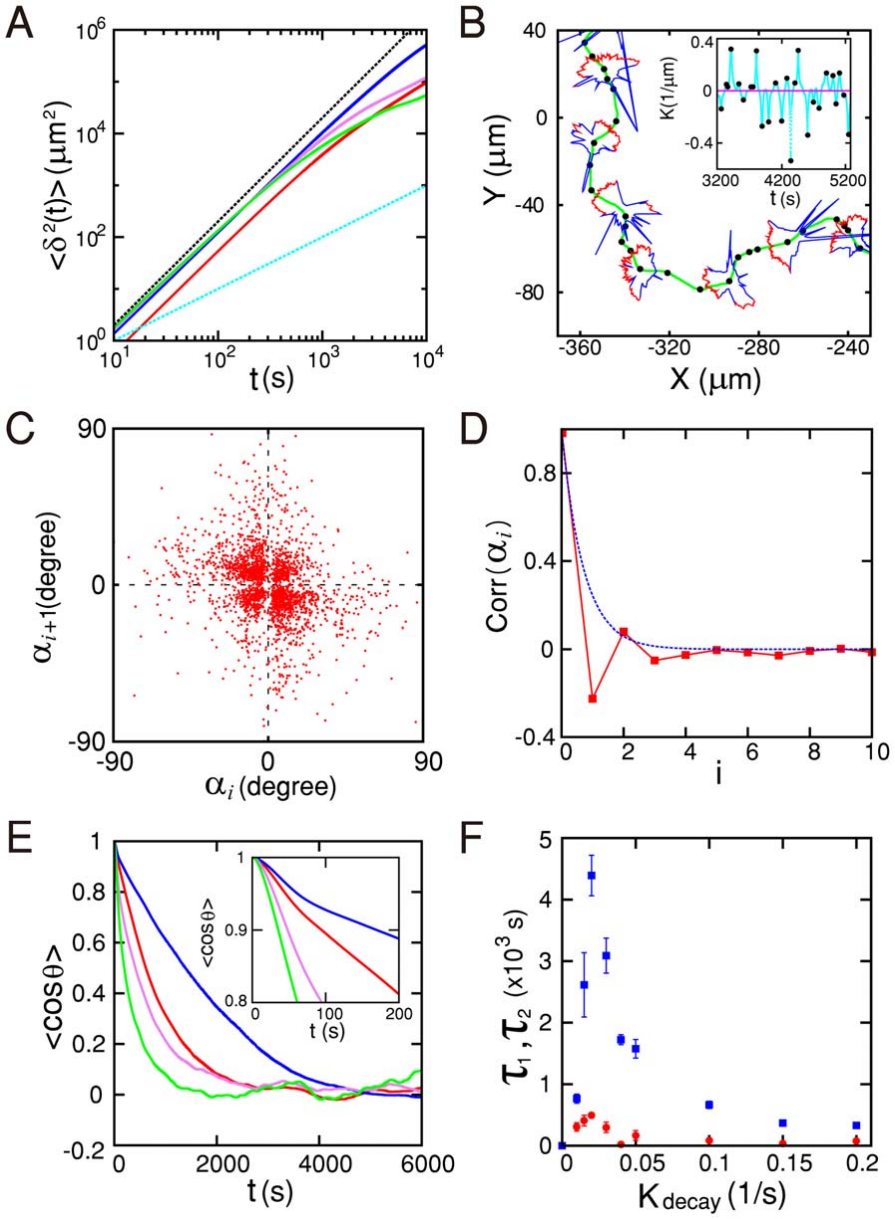
Long-range directional persistence and zigzag turns of the crawling trajectories of a mathematical model cell. A) Mean square displacements vs. time for 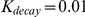 (red), 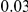 (blue), 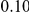 (violet), and 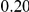 (green). The cyan and black dotted lines have a slope of 1 and 2, respectively. B) Close-up view of the green highlighted segment in Fig. 3 (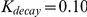). Some snapshot images of the crawling cell are superimposed on the trajectory. The red (blue) boundary is the moving front (trailing edge) where 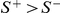 (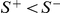). The inset plots the instantaneous local curvature along the centroid trajectory. Local maxima and minima are marked by red dots, which correspond to the turning points (black dots) along the centroid trajectory. C) Return map of the turning angle (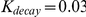). The zigzag preference 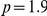. D) Auto-correlation function of the sequence of turning angles (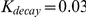). The blue dotted line is an exponential function fit with a decay time constant of 0.705. E) Auto-correlation functions of the instantaneous direction of movement for 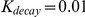 (red), 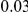 (blue), 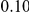 (violet), and 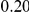 (green). F) Two time constants obtained by fitting 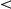
cos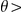 to 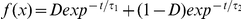. The error bars represent the standard deviation based on 10 different trajectories obtained with a different initial condition.

Figure 3 shows four different model cell trajectories obtained by changing one of the key parameters, 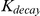, which is the decay rate constant of 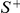. The directional persistence of the model cell trajectory varies significantly as a function of 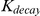 (see Fig. 4A). For example, for 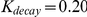 the linear regime ends below 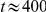, whereas for 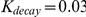 it extends over 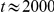. The visual similarity of the model cell (Fig. 4B) to the real microglia (Fig. 1A) is quite striking. Moreover, the trajectories of the model cell can also be viewed as a sequence of small ‘runs’ and ‘turns’ as shown in Fig. 4B. The probability distribution of turning angles 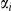, inter-turn distances 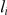, inter-turn time intervals 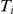, and inter-turn average velocities 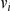 (see Fig. S4) are similar to those obtained in the experiments with PMG and MG5 cells (see Fig. S2). Occasionally, the model cell also shows front-splitting events as shown in Fig. S3 similar to the case of the PMG cell. In addition, the model cell trajectories also favor zigzag turns, which are again well captured in the return map of 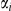 (see Fig. 4C). Moreover, the auto-correlation function of 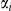 (Fig. 4D) shows that the memory of the last turn affects only the current turn and decays quickly thereafter. As in the case of microglia crawling trajectories, the function 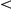
cos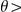, measuring the directional persistence, of the model cell was well fitted to a sum of two exponential functions (Fig. 4E). Again, 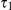 and 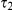 correspond to the two time scales, one for the small ‘runs’ and the other for the long-range directional persistence. The measured value of 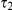 changes significantly (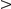
10 fold) as 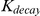 changes from 0.01 to 0.20 with its maximum value near 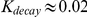 as shown in Fig. 4F.

**Figure 5:**
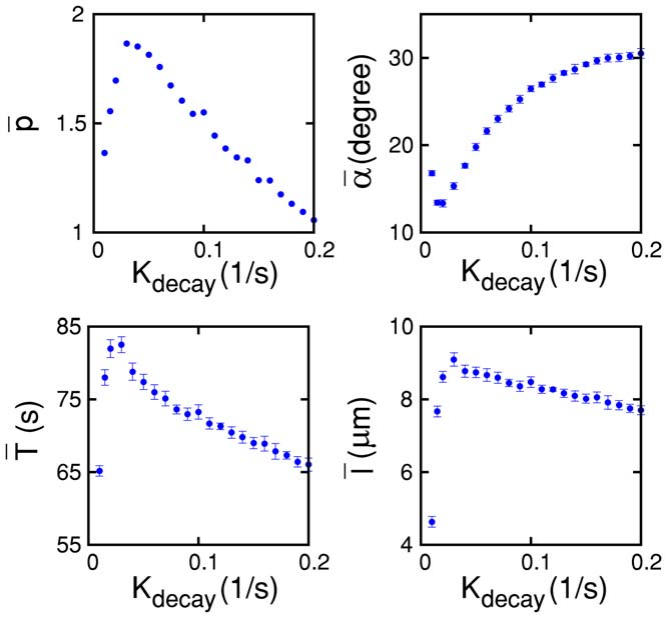
Various properties of the model cell trajectories: A) mean zigzag preference factor, B) mean turning angle, C) mean inter-turn time interval, and D) mean inter-turn distance. The error bars indicates the s.d. for 10 different trials with a different initial condition.

Changing 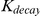 affects a number of different features of the cell trajectory simultaneously as shown in Fig. 5. As 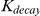 changes from 0.01 to 0.20, 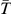, 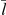, 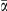, and 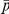, change by 26.6%, 96.3%, 128.8%, and 76.6%, in that order, with respect to their corresponding minimum values. The minimum of 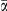, the maxima of 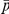, 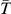 and 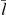 all are closely located around 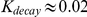. Of course, the smaller 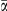 is or the larger 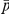, 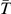 and 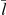 are, the larger 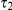 becomes. Therefore, in the simulations 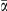, 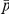, 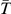 and 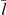 all contribute synergetically to the maximum peak of 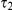 near 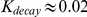.

## Discussion

The moving trajectories of the freely crawling rat microglia and mouse-derived microglia cell line, which had been grown in culture for 2–4 days, were analyzed carefully by long-term time-lapse video imaging. The trajectories could be viewed as a chain of small ‘runs,’ and in many cases two successive angles connecting the runs along the chain were not random but anti-correlated. This anti-correlation is statistically significant. In addition, similar behavior was identified in a simple rule-based model cell that incorporates the biochemistry of actin polymerization/depolymerization of the cell shape regulation. The properties of the model cell trajectories, as characterized by 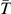, 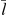, 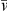, 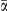, 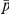, 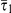 and 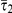, could be changed significantly by varying the decay rate 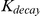 of the activator species associated with actin polymerization. These findings are consistent with those reported by Li *et al.* [15] regarding the motility of starving Dictyostelium amoebae. Table 1 lists various quantities of dicty cells as well as PMG, MG5, and the model cell.

Soon after Li's work on dicty cell motility, Maeda *et al.* [16] published an interesting article on a closely related issue. They examined the dynamics and statistics associated with the cell shapes of crawling dicty cells in their moving frames. They identified three distinct dynamical states, elongated, rotating, and oscillating, and found that it was typical of crawling dicty cells to have abrupt transitions among these different states. No detailed statistics regarding the time intervals associated with the transitions were provided. The period of oscillation for the oscillatory states was typically 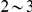 min, which is similar to what Li *et al.* reported for the mean inter-turn time interval (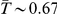 min). The oscillatory state can be viewed as an unusual case in which all successive turns result in a quite regular zigzag pattern, even though Li's work reported that the dicty memory is only short-range rarely extending over one turn. This contradiction can originate from the difference in the characteristics of the cells being investigated or in the environments to which the cells were subjected to.

Regarding the regular oscillatory behavior of crawling cells, Barnhart *et al.* [1] reported a robust “bipedal locomotion” of crawling fish epithelial keratocytes. They found persistent oscillatory movement in which retraction of the trailing edge on one side of the cell body is out of phase with retraction of the other side. In other words, the trailing edge oscillation is the key for keratocyte locomotion, whereas the front dynamics is believed to be the key component in the navigation of crawling amoebae and microglia. The authors also provided a mathematical model viewing the keratocytes as a three-component stick/slip elastic system, in which a leading front is coupled mechanically to the left and right portion of the tailing edge. Nonlinear elastic coupling between the front and tail was the key for rendering the lateral periodic oscillation of the cell body. One important result of their model is the positive correlation between the mean cell speed and oscillation frequency. As an analogy, PMG and MG5 cells also show a positive correlation between the average cell speed and the inverse of the average inter-turn time interval (not shown).

Directional persistence of crawling cells was also discussed in several other recent reports. For example, Selmeczi *et al.* [25] investigated the motile patterns of human keratincocytes and fibroblasts, and found that some key properties of much studied OU model conflicted with their experimental data. With an extra term, which carries the memory of past velocities, added to the simple OU model, however, they could better describe the trajectories of the cells. Another study on the crawling behavior of freely moving cells was reported recently by Dieterich *et al.* [6]. They observed anomalous cell migrations of renal epithelial Madin-Darby kidney cells and reported that their crawling paths were best described by the fractional Klein-Kramers equation which involved temporal memory. Once again, it was indicated that neither the worm-like chain model nor the simple OU model were suitable for describing the crawling cells of their concern. The mathematical models proposed by Selmeczi *et al.* or Dieterich *et al.* may also be applicable to dicty amoebae and microglia, since both models have components for data-driven tailoring of cell-specific type. These two reports, however, do not specifically discuss the zigzag turning behavior of crawling. But, we indicate that the moving trajectory of the epithelial cell reported in [6] could also be viewed as a sequence of ‘run-and-turns’ showing a strong tendency of zigzag turns.

In summary, the trajectories followed by freely crawling cells are viewed as a chain of ‘run-and-turns, ’ and then the cells appear to favor making zigzag turns. This contrasts with the well-known bacterial tumbling that results in the random selection of a moving direction. The cultured microglia of rat brains and MG5 cell lines as well as Dictyostelium amoebae are good examples of this zigzag motility hypothesis. Satulovsky's simple mathematical model cell incorporating only the essential biochemistry of actin polymerization and cell mechanics also generates a similar motile behavior. Taken all together, the observed zigzag turning behavior is believed to be a generic feature of many different crawling cells in isolation. The key biophysics that underlies the observed inherent zigzag motility might be due to the extension pattern of pseudopodia [4] and the spatiotemporal dynamics of the coherent actin waves [29]. However, the generality of the observed motile behavior and the underlying mechanisms need to be further tested using many other types of cells and models. Finally, we should indicate that the run-and-turn chain scenario is an interpretation that is forced on smooth trajectories of crawling cells: the trajectories themselves are not piecewise linear but differentiable. The main finding of this investigation is that crawling cells seem to prefer to have zigzag motility with a short-term memory, and our analyis leading to this conclusion does not require that the cell trajectories to be a piecewise linear chain.

## Materials and Methods

### Ethics Statement

All experimental procedures and protocols were in accordance with the guidelines established by the Committee of Animal Research Policy of Korea University College of Medicine.

### Microglia cell culture

Primary glia co-cultures were prepared from the cerebral cortex of postnatal day 1–2 Sprague Dawley rat brains (Charles River, OrientBio Inc.). The brains were excised quickly and the cerebral cortices were removed and cleared of meninges under a dissecting microscope. After a papain (6 unit) treatment (10 min), fragmented cortical tissues were collected and dissociated mechanically using a fire-polished Pasteur pipette in 2 ml of DMEM supplemented with 10% FBS. The dissociated cells were grown in T-75 culture flasks (BD Falcon) (37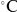, 5% CO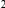) with an initial seeding density of 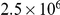 cells/flask in 10 ml DMEM with 10% FBS. The culture medium was changed in every 5 days.

After growth in T-75 flasks for several days, the cells were shaken with new culture media at 120 rpm for 10 min to remove the dead cells, and subsequently shaken at 280 rpm for 20 min to harvest the microglia cells detached from the substrate. The supernatant was collected, centrifuged for 5 min at 1500 rpm, and the microglia cells thus acquired were plated on a cover-slips (50 cells/mm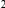) for observation. After plating, the cells were stabilized in an incubator (37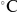, 5% CO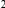) for three hours, and the culture media was gently replaced with DMEM with 10% FBS to further remove the dead cells. The same culture medium and protocol used for the PMG cells after dissociation was used for MG5 cell culture. The MG5 cell line was a gift from Dr. Ikeda at Toyama University, Japan.

### Time-lapse imaging and data processing

Culture dishes containing the PMG (or MG5) plated cover-slips were placed in a temperature 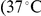) and CO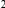 (5%) regulated home-built chamber mounted onto the stage of an inverted microscope (IX71, Olympus) with an objective lens (20x, NA 0.55). Time-lapse images were acquired at 15 sec intervals, typically for a time period longer than 24 hours, using a cooled CCD camera (MFcool, ProGres) with a spatial resolution of 0.5 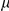
m/pixel. To trace out the trajectory of a crawling cell and identify its turning events, the acquired images were binarized using the ImageJ program and the centroid of the cell body was calculated for each frame. We have assumed that the mass density is uniform. The sequence of the centroid positions (X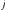,Y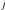) [green dots in Fig. S5A] was low-pass filtered by locally fitting them separately to a third order polynomial function with a sliding window of 11 successive points [red solid lines in Fig. S5B and S5C], which corresponds to 150 sec in time or 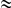
7 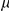
m in space. The local curvature 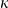 was calculated at each time step using the fitted functions [blue dots in Fig. S5D]. Finally, a lowpass filter was applied to 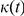 with a cutoff at 30 sec, and the local extrema of the filtered 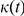 [black dots in Fig. 5D] were considered to be a turning point.

### Fitting and filtering effects on the zigzag preference

Whether it is an experimental data or a simulation result, the centroid trajectories of crawling cells have some high-frequency fluctuation due to intrinsic physiological noise as well as errors in the imaging and data processing. Then, the questions are how much do we smooth the raw data before we identify a turning event as an extremum of the local curvature 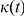. As discussed in the previous section, the smoothing process involves 1) a local fitting of the centroid positions to a third order polynomial function and 2) a lowpass filtering of 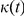. Naturally, the zigzag preference 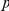 depends on the cutoff values that we introduced during the smoothing process.

The size of the fitting window significantly influences the number of turning events as shown in Fig. S6 and S7A: with the smaller window size, the larger the number of turns becomes. The temporal range (40
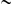
160 sec) that we have explored in Fig. S7A corresponds to the spatial range of 4.2
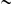
16.8 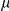
m approximately, considering that the mean crawling velocity is about 6.3 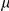
m/min for the given set of parameter values. This is a physical range in which the zigzag phenomenon is relevant since the size (diameter) of the model cell is about 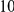 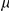
m. In other words, we are only interested in the zigzag turns arising more or less at the physical size of a single cell. Since the local fitting process has a spatial lowpass filtering effect, a window size that is too small will results in too many uncorrelated noisy turns as shown in Fig. S7B. On the contrary, a window size that is too big will wipe out the zigzag information. Figure S7C plots the effect of the fitting window size on the zigzag preference: it varies significantly but the zigzag turning preference remains over the whole range being explored. After the local curvature 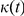 was computed with the smoothed cell trajectories, a (boxcar) lowpass filter was applied to 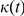 to exclude very small-angle high-frequency turns. The effect of the filter size on the total number of turns and the zigzag preference is plotted in Fig. S8. Again, they vary but the zigzag phenomenon remains the same. Our analysis on the model cell trajectories given in Fig. 4 and Fig. S4 are based on the fitting window size of 101 sec and the lowpass filter size of 21 seconds.

### Mathematical crawling cell model

The mathematical model cell proposed by Satulovsky *et al.* [24] was simulated to determine if it could exhibit the zigzag motile behavior observed in the current experiments. A crawling cell was modeled as a simply closed contour on a two-dimensional space, which evolves in space and time. The points along the cell perimeter are represented as vectors 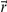 with the centroid of the cell being the origin. The concentration of the activator 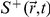 is a local variable, whereas the concentration of the inhibitor 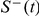 is a global variable. At each iteration time step, each point along the perimeter can either advance, retreat, or does not move based on the following set of rules. Retraction occurs when 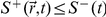, and the rate of retraction is governed by the following stochastic equation:
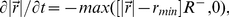
(1)where 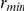 is the constant minimum radius and 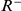 is the retraction rate constant. The function 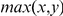 selects the larger value of 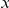 and 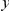. Protrusion occurs when 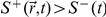 at a rate governed by the following equation:
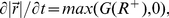
(2)where 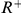 is the average protrusion rate and 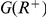 is a random number generated from a Gaussian distribution of the mean 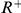 and variance 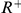. The evolution of activator 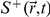 is governed by the equation
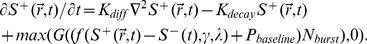
(3)The first two terms are deterministic, whereas the third term is a stochastic positive feedback loop accounting for both the local stimulation and the existence of a random signal. The function 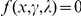 for 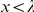 and 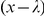 for 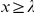, where 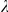 is a threshold value for the feedback. 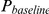 accounts for the rate of random bursts cased by internal baseline activities. The function 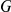 again represents a random number generated from a Gaussian distribution. The retraction signal is governed by a global inhibition rule 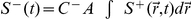, where 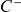 is the inhibition constant, 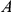 is the total area of the cell, and the integration is a line integral over the entire cell border, which is composed of 360 pixels (i.e., 1 pixel for 1 degree with respect to the centroid). Each pixel corresponds to 0.286 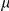
m and one iteration time step is one second. At each iterative time step, the formation of focal adhesions and their detachments are assigned stochastically to the points along the cell perimeter with a probability 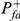 and 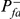, respectively. Retraction is inhibited when a perimeter point hits a focal adhesion. The biophysical justifications for the above set of equations and the numerical iteration scheme are described in detail in reference [24]. The values of the eleven parameters 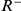, 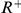, 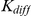, 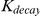, 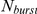, 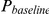, 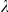, 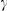, 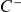, 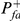 , 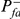 used for the numerical simulations are specified in the figure caption of Fig. 3.

## Supporting Information

Figure S1Mean velocity distribution of a PMG cell for different values of 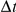.(TIF)

Figure S2Probability density functions associated with the trajectories of PMG (red) and MG5 (blue) cells: A) turning angle, B) inter-turn distance, C) inter-turn time interval, and D) inter-turn mean velocity (error bar: SEM, n = 8 for each case). The two straight lines in (C) are an exponential function fit for 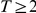 min: PMG (slope = −2.9), MG5 (slope = −3.5).(TIF)

Figure S3Sequence of snapshot images showing a ‘front-splitting’ event: A) PMG cell and B) the model cell (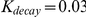). Each frame is 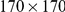 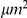 for (A) and 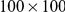 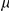
m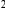 for (B). The green lines in (B) represent the path of the centroid.(TIF)

Figure S4Probability density functions associated with the trajectories of the model cell: A) turning angle, B) inter-turn distance, C) inter-turn time interval, and D) inter-turn mean velocity [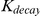 = 0.01 (red), 0.03 (blue), 0.10 (violet), and 0.20 (green)]. The straight line in (C) is an exponential function fit for 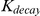 = 0.03 for 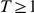 min (slope = −1.26).(TIF)

Figure S5Defining turning points: A) a PMG cell trajectory (raw data: green, smoothed data: red) with turning points marked by red dots, B) x-coordinates in time, C) y-coordinates, and D) local curvature 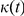 computed with the fitted values of 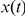 and 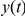 shown in (B) and (C). In (D), turning points are marked by black dots. This trajectory data matches the supplementary Movie S1.(TIF)

Figure S6Fitting window size effect on the number of turns: (green) raw trace of model cell trajectory for 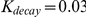, (black and red dots) turning points obtained with a fitting window size of 101 sec and 51, respectively. Inset: blown-up image of the boxed area. The black line within the inset is the smoothed trajectory obtained with the fitting window size of 101. The result is based on 200000 seconds of iteration.(TIF)

Figure S7Fitting window size effect on the zigzag preference factor 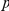: A) Number of turns vs. fitting window size, B) Return maps of turning angle sequences, C) 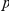 vs. fitting window size.(TIF)

Figure S8 Filter cutoff size effect on the number of turns (dots) and the zigzag preference (square).
(TIF)

Movie S1 A time-lapse movie showing a freely crawling rat microglia cell: (green line) smoothed centroid trajectory and (red dots) turning points.
(WMV)
