# Supplementary figures and images for: Zigzag Turning Preference of Freely Crawling Cells
Source: PLoS One. 2011 Jun 7;6(6):e20255. doi: 10.1371/journal.pone.0020255 (PMC3110194; doi:10.1371/journal.pone.0020255)

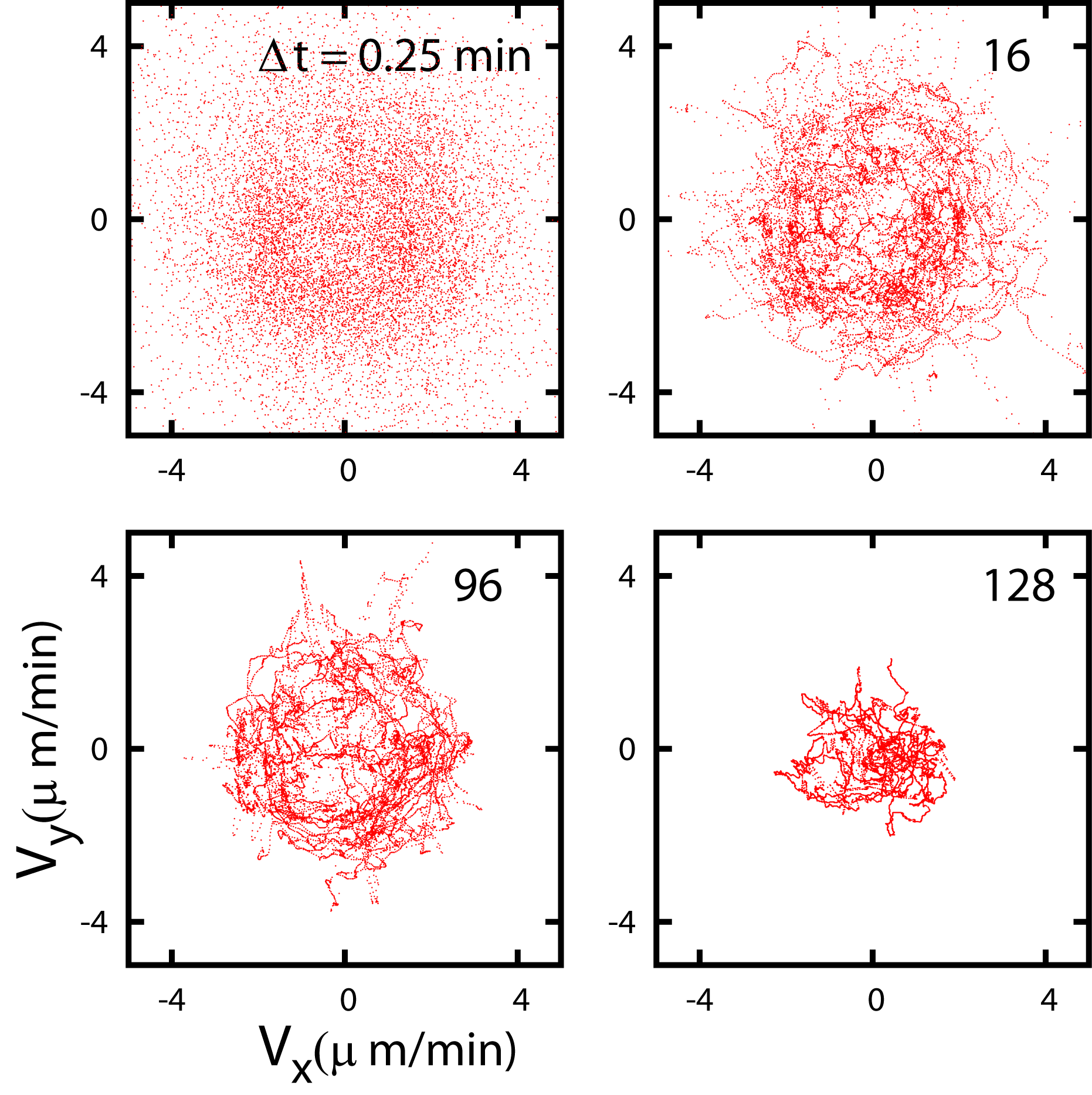

Supplement: Figure S1 — Mean velocity distribution of a PMG cell for different values of . (TIF) [file pone.0020255.s001.tif]

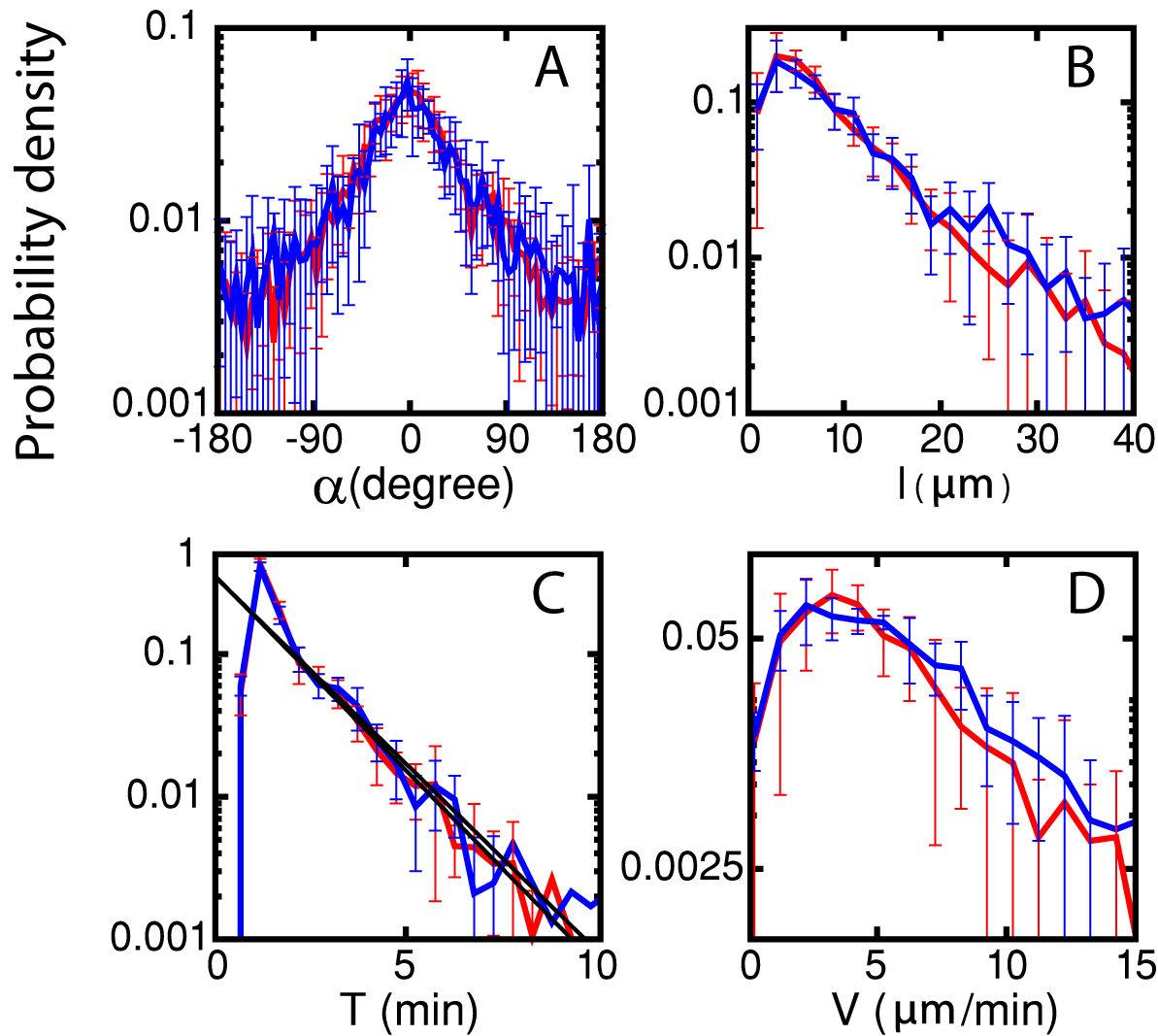

Supplement: Figure S2 — Probability density functions associated with the trajectories of PMG (red) and MG5 (blue) cells: A) turning angle, B) inter-turn distance, C) inter-turn time interval, and D) inter-turn mean velocity (error bar: SEM, n = 8 for each case). The two straight lines in (C) are an exponential function fit for min: PMG (slope = −2.9), MG5 (slope = −3.5). (TIF) [file pone.0020255.s002.tif]

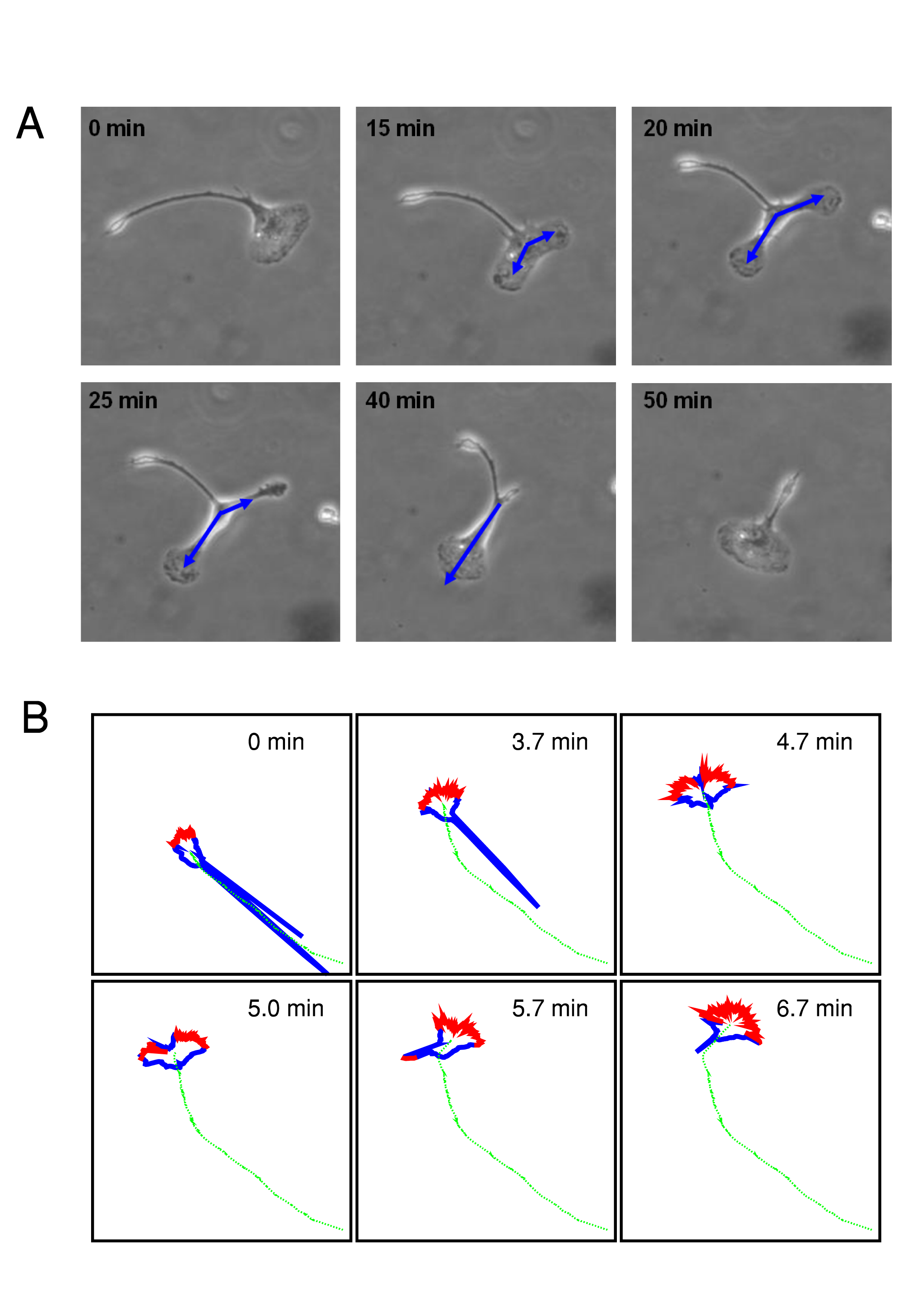

Supplement: Figure S3 — Sequence of snapshot images showing a ‘front-splitting’ event: A) PMG cell and B) the model cell (). Each frame is for (A) and m for (B). The green lines in (B) represent the path of the centroid. (TIF) [file pone.0020255.s003.tif]

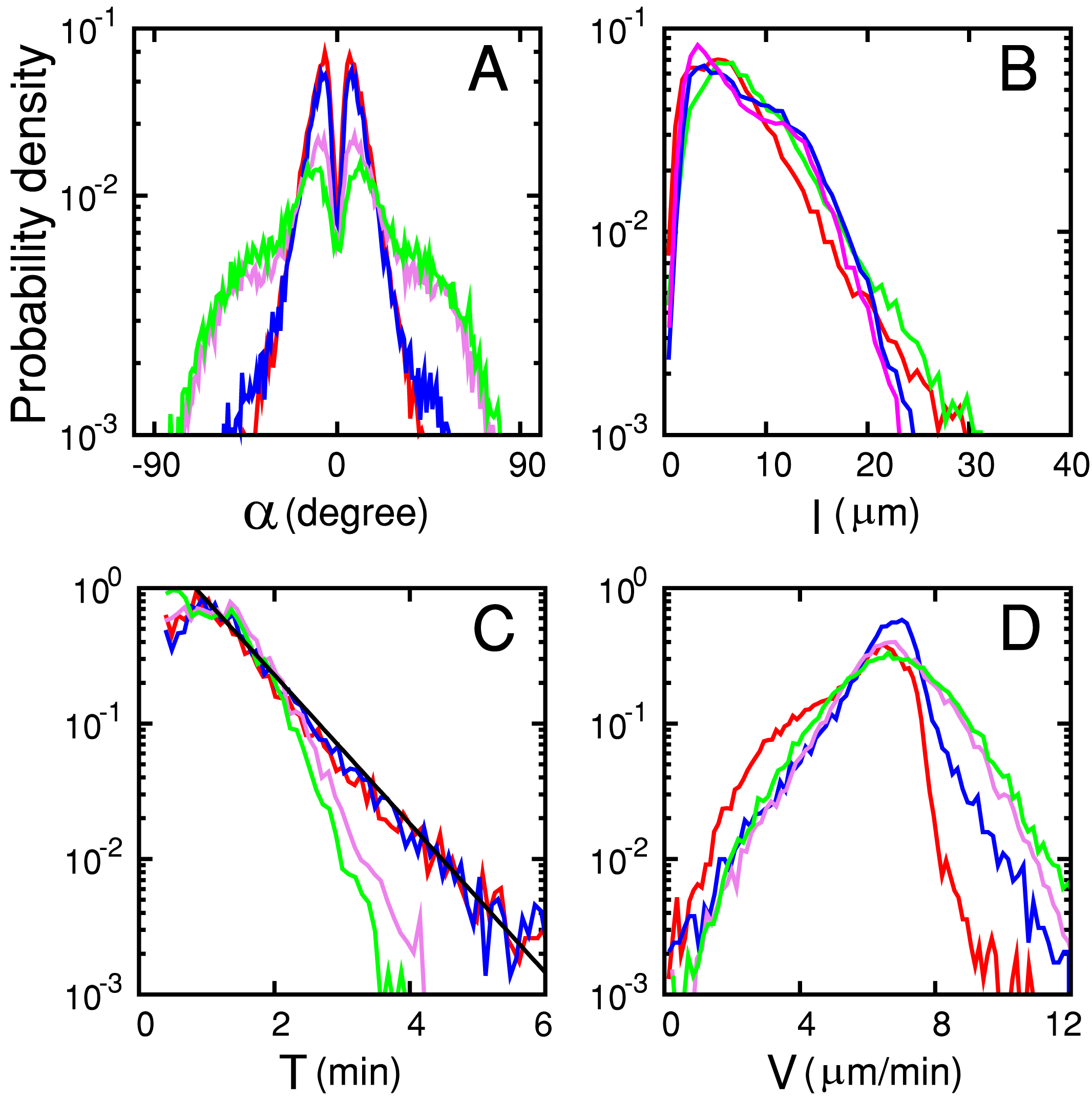

Supplement: Figure S4 — Probability density functions associated with the trajectories of the model cell: A) turning angle, B) inter-turn distance, C) inter-turn time interval, and D) inter-turn mean velocity [ = 0.01 (red), 0.03 (blue), 0.10 (violet), and 0.20 (green)]. The straight line in (C) is an exponential function fit for = 0.03 for min (slope = −1.26). (TIF) [file pone.0020255.s004.tif]

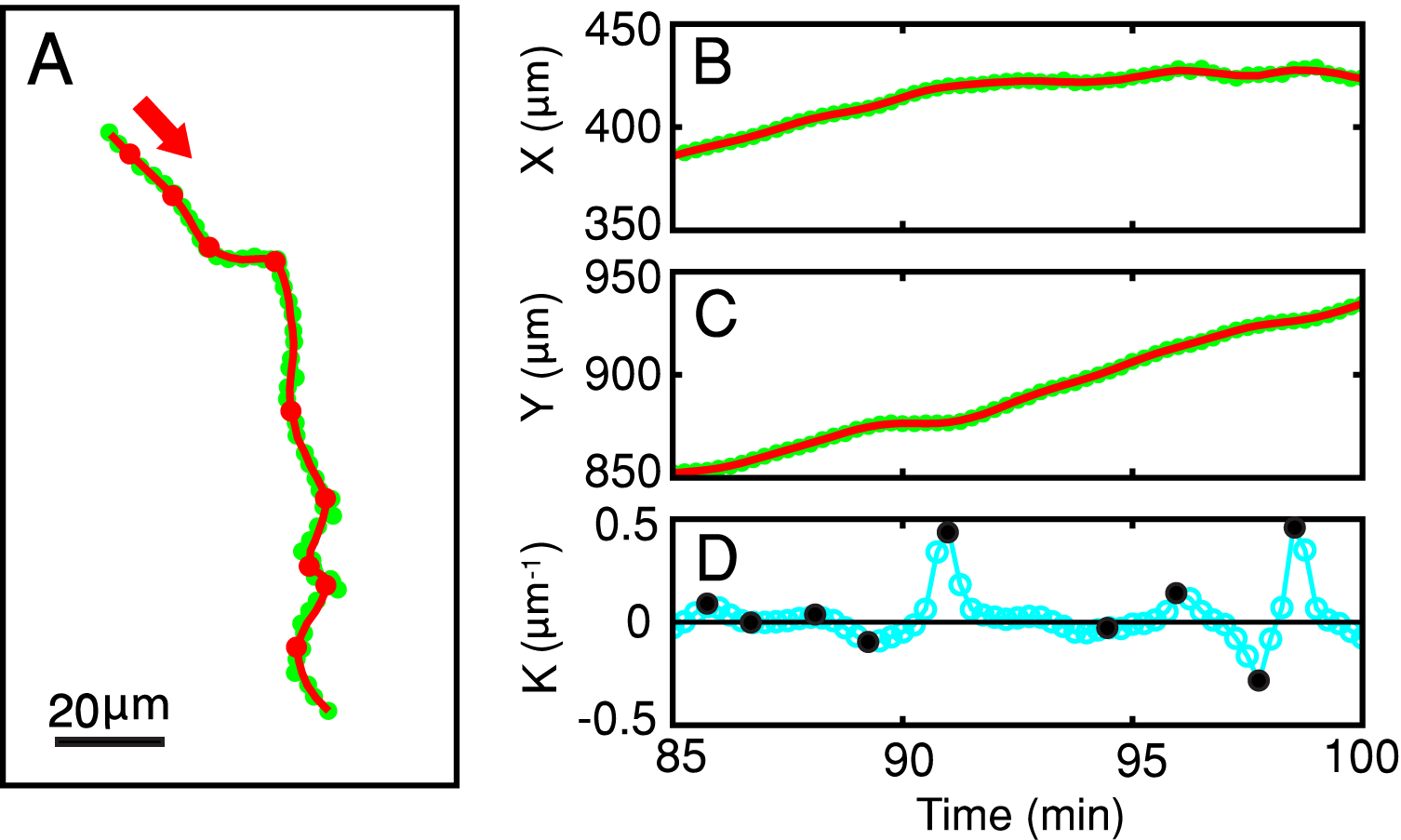

Supplement: Figure S5 — Defining turning points: A) a PMG cell trajectory (raw data: green, smoothed data: red) with turning points marked by red dots, B) x-coordinates in time, C) y-coordinates, and D) local curvature computed with the fitted values of and shown in (B) and (C). In (D), turning points are marked by black dots. This trajectory data matches the supplementary Movie S1. (TIF) [file pone.0020255.s005.tif]

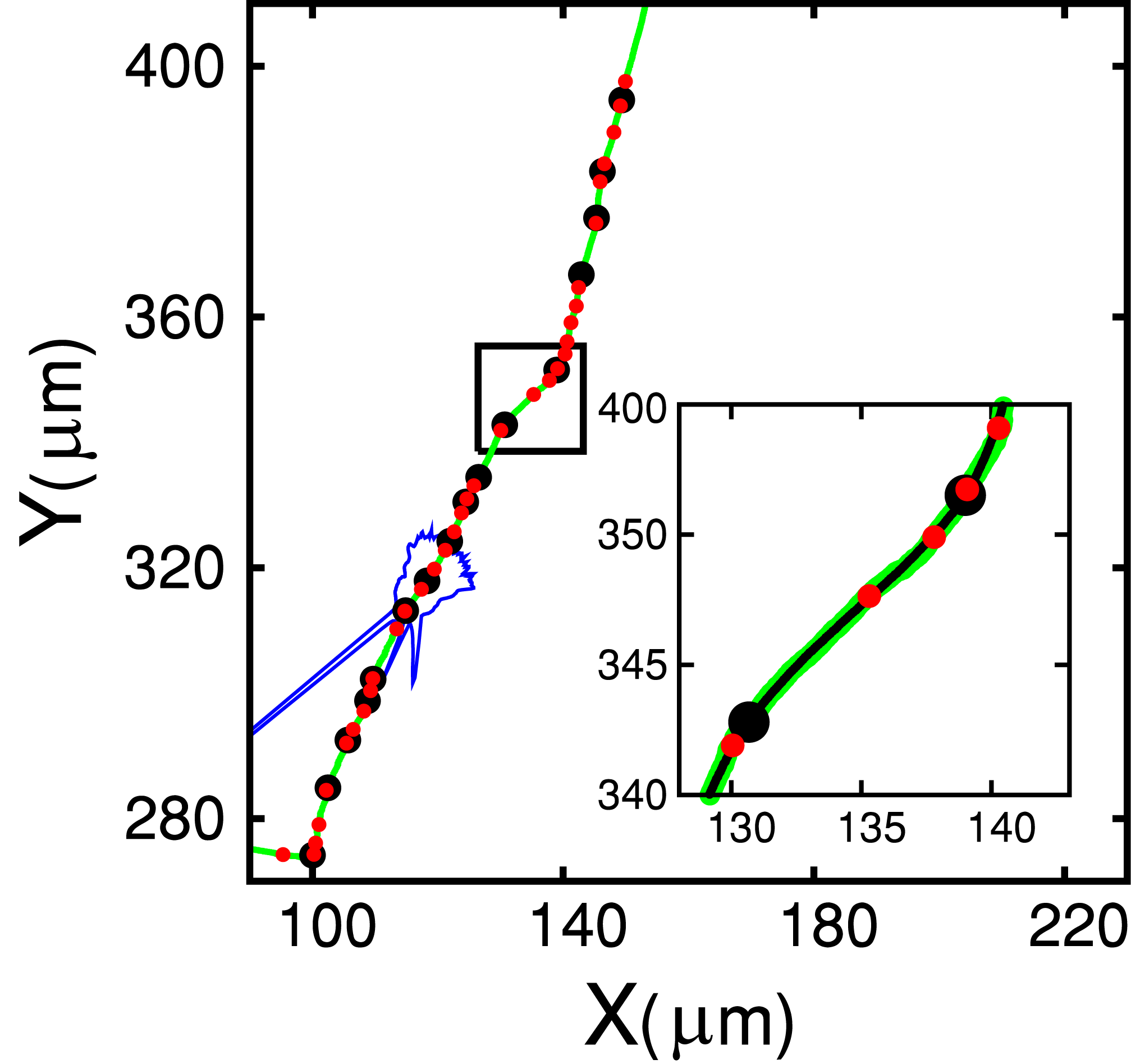

Supplement: Figure S6 — Fitting window size effect on the number of turns: (green) raw trace of model cell trajectory for , (black and red dots) turning points obtained with a fitting window size of 101 sec and 51, respectively. Inset: blown-up image of the boxed area. The black line within the inset is the smoothed trajectory obtained with the fitting window size of 101. The result is based on 200000 seconds of iteration. (TIF) [file pone.0020255.s006.tif]

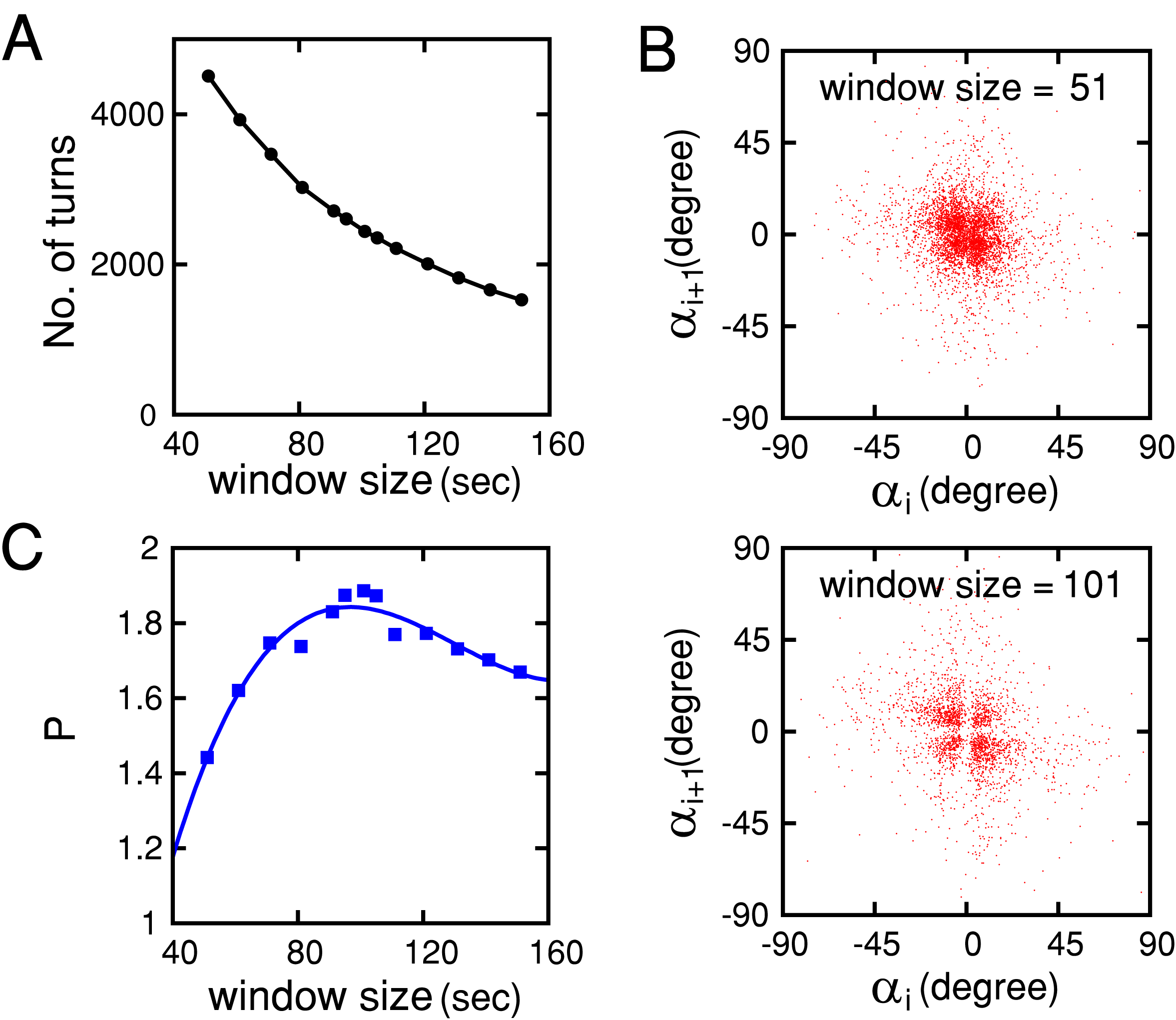

Supplement: Figure S7 — Fitting window size effect on the zigzag preference factor : A) Number of turns vs. fitting window size, B) Return maps of turning angle sequences, C) vs. fitting window size. (TIF) [file pone.0020255.s007.tif]

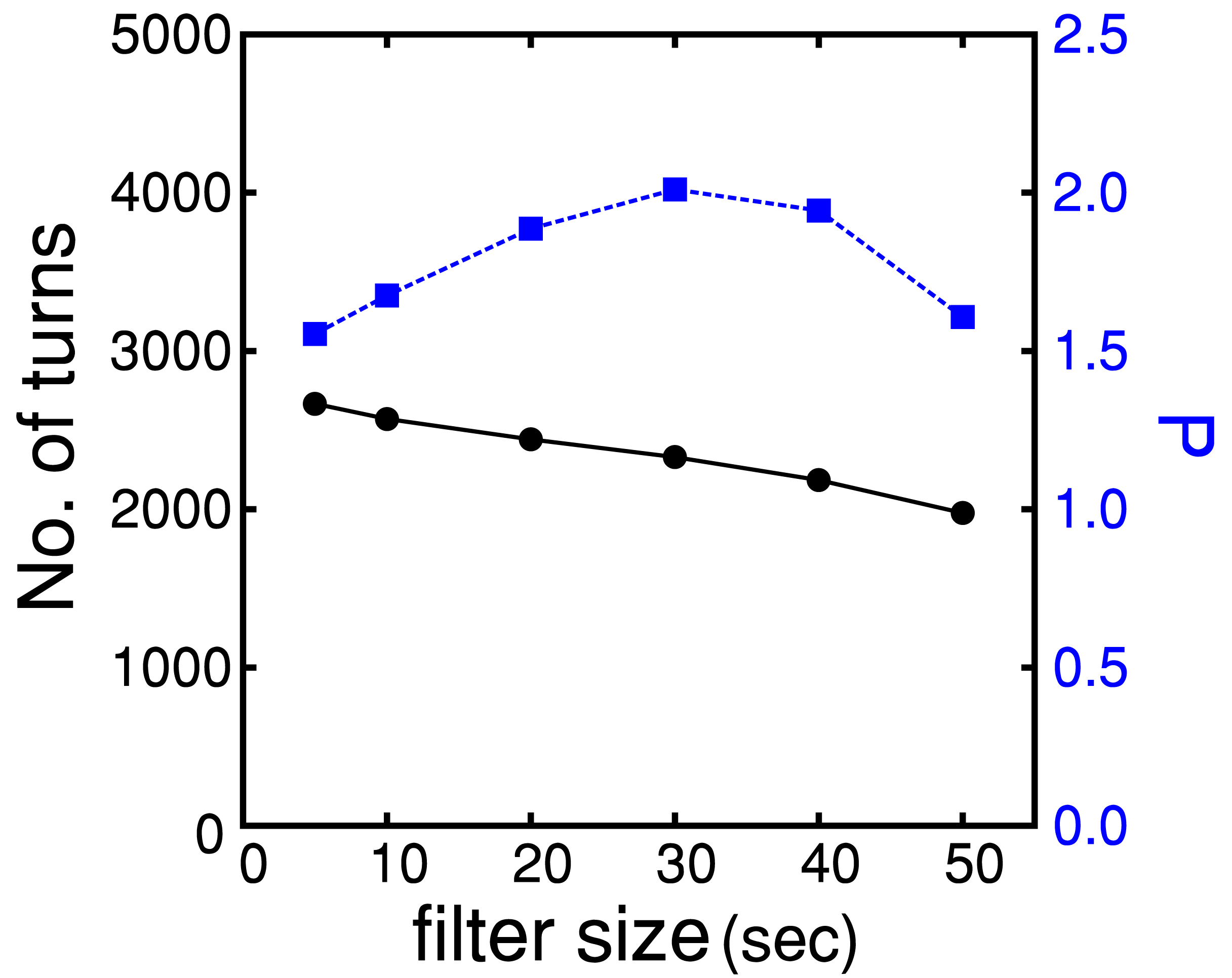

Supplement: Figure S8 — Filter cutoff size effect on the number of turns (dots) and the zigzag preference (square). (TIF) [file pone.0020255.s008.tif]
